# The Treatment of Whooping-Cough—I

**Published:** 1909-04-03

**Authors:** 


### Diseases of Children.

### THE TREATMENT OF WHOOPING-COUGH—I.

It is decidedly disappointing that there has been so little improvement in the methods of treating whooping-cough, in spite of the enormous strides in the science of bacteriology, in serum therapeutics, vaccines, and the preparation of synthetic drugs. The recent discussion on the subject at the Royal Society of Medicine shows that the number of remedies is multiple, but the value assigned thereto by different authorities small.

The treatment ought to be based on the pathogeny and the morbid anatomy; but here again we tread upon uncertain ground. A general hyperæmia and congestion of the whole of the respiratory tract, from the posterior nares to the bifurcation of the trachea, is present. The posterior wall of the pharynx between the vocal cords and the under surface of the epiglottis are the most affected. During a paroxysm a small pellet of mucus may be seen on the posterior wall of the larynx at the level of the glottis, and removal of this stops the paroxysm (Von Herff, 1887). Irritation of this part causes a coughing fit, whereas other parts can be touched with impunity. Examination with a spatula may cause reflex irritation of the larynx and a paroxysmal cough; so, too, a crumb going the wrong way.

These observations suggest that the whooping depends on local conditions. Possibly it is influenced by the effect of the specific toxin on the nervous system, for other forms of laryngo-tracheitis do not set up the characteristic cough unless the child has previously had whooping-cough. The actual paroxysm can be stopped at the onset by dashing cold water in the child's face. This must be the result of a sudden inhibition due to shock. It can also be stopped by making the child vomit, as by inserting the finger into the throat. Here the result is almost certainly due to the act of vomiting enabling the child to get rid of the viscid, tenacious, irritating pellet of mucus. Naegeli recommended that the lower jaw should be pulled downwards and forwards. Sobel found this method successful in 87 out of 96 cases between the ages of three months and eight years, and that it is more successful in the older children. It must not be used when there is food in the mouth or œsophagus. It may overcome the expiratory spasm and asphyxia in cases without whooping.

The general treatment must be directed to the maintenance of the general health, the prevention of inanition due to severe vomiting, the guarding against pulmonary complications, the reduction in the frequency and the intensity of the paroxysms, and the shortening of the duration of the disease. The supply of fresh, pure, mild air must be as liberal as possible, though dependent on climatic conditions. The child should be kept in bed if there is much debility or a raised temperature. Two rooms should be available and used alternately. The temperature should be kept at 55° F. to 60° F., and 5° higher in infancy or if there are complications. There is no necessity for confinement to bed or to the house if the weather is warm and dry and the disease is uncomplicated. Under such circumstances open-air treatment throughout is the best plan. Dust and draughts must be avoided. The diet must consist of soft, light, easily digestible food which does not irritate the pharynx and is not liable to set up flatulent distension of the stomach. If there is much vomiting, small frequent meals should be given after the paroxysms. It may even be advisable to induce vomiting before feeding. The state of the stomach and intestines must be attended to. An occasional dose of grey powder or rhubarb and soda is decidedly beneficial. The bowels should be kept freely open.

An abdominal belt, worn day and night, is comforting, prevents vomiting, and reduces the paroxysms. It should be made of linen or flannel, four to eight inches wide, three inches less in length than the girth at the navel, with an insertion on each side of two inches of elastic webbing or stockinette, and lacing up the back. It must be applied moderately tightly over the binder and under the vest, avoiding any compression of the thorax. It is often advisable to remove adenoids if they are present, especially if they interfere in the very least with respiration. The naso-pharynx should be kept disinfected with some mild disinfectant or douched morning and evening with Dobell's solution.

Serum has been prepared by Leuriaux and by Manicatide. The former inoculated horses with broth cultures of his bacillus, and claims good results from injections of five cubic centimetres. Several observers have noted that vaccination has a beneficial effect on the severity and duration of the disease.

On the assumption that the affection is due to local inflammation of the nose, pharynx, or larynx, various local applications have been vaunted as beneficial. Certainly it is advisable to keep the nose and throat as clean as possible, as a prophylactic measure against secondary infections. They may be sprayed with lotions of common salt, bicarbonate of soda, boric acid, or hydrogen peroxide in glycerin and water. As a specific the nose may be irrigated with a 2.5 per cent. warm solution of carbolic acid.

For insufflation or as an ointment for insertion into each of the nostrils one may use bismuth salicylate 5 parts, benzoin 5 parts, quinine sulphate 1 part; or boric acid 10, menthol 5, vaseline 30 parts. Insufflation of powders into the larynx is injurious and likely to induce spasms. The fauces may be painted once daily with a solution of one in a thousand corrosive sublimate as an antiseptic, or with a 5 per cent. solution of cocaine to stop vomiting and spasm; or a 2.5 per cent. solution of resorcin may be applied with a fine sponge to the epiglottis every four hours. Obviously many of the above applications are only suitable for older children. The further measures of treatment by inhalations, counter-irritants, and internal medication will be considered in another article.

